# Acetabular Liner Dissociation following Total Hip Arthroplasty: A Rare but Serious Complication That May Be Easily Misinterpreted in the Emergency Department

**DOI:** 10.1155/2015/802753

**Published:** 2015-10-12

**Authors:** Christopher K. J. O'Neill, Richard J. Napier, Owen J. Diamond, Seamus O'Brien, David E. Beverland

**Affiliations:** Primary Joint Unit, Musgrave Park Hospital, Stockmans Lane, Belfast BT9 7JB, UK

## Abstract

Acetabular liner dissociation is a rare complication of Total Hip Arthroplasty (THA) which requires urgent revision surgery. A case is presented in which the correct diagnosis was not appreciated on two separate Emergency Department attendances. The typical symptoms, signs, and radiological features are outlined and the importance of considering a rare complication following a commonly performed procedure is highlighted.

## 1. Introduction

Total Hip Arthroplasty (THA) remains one of the most frequently performed orthopaedic procedures performed worldwide [1–3]. Surgery involves implantation of both a femoral and acetabular component, with options for either cemented or uncemented fixation depending on Surgeon preference. The modern uncemented acetabular component is modular in design and provides several theoretical advantages. One of the primary attractions is the ability to implant alternative bearing surfaces with improved wear characteristics. The modular design also permits certain conservative revision options in the event of early infection, late polyethylene wear, or recurrent dislocation [4].

## 2. Case Presentation

An 83-year-old male was referred to Orthopaedic Outpatient Clinic by his General Practitioner (GP) for further assessment of a painful right THA.

The uncemented THA (Corail KLA12 stem, Pinnacle 100 Series 54 mm sell, 54/28 mm highly cross-linked polyethylene Marathon liner: DePuy, Warsaw, IN, USA) was performed five years previously for osteoarthritis. The patient had no other significant medical comorbidity. Body Mass Index was 27.4 kg/m^2^ and activity level was relatively low demand.

Surgery was performed in the lateral decubitus position via a posterior approach, using the Transverse Acetabular Ligament (TAL) as a reference to control acetabular component orientation. Acetabular component radiological inclination (RI) and radiological anteversion (RA) were within the desired range (RI 45.1°, RA 10.7°).

The THA was a well-functioning implant until approximately 4 months prior to referral. At that time, the patient reported sudden onset of severe groin pain when getting into bed and attended the Emergency Department (ED) for assessment.

No significant limb length discrepancy or neurovascular deficit was documented. X-rays were interpreted as satisfactory, with no evidence of fracture or dislocation, and the patient was discharged.

The groin pain improved over a two-week period and the patient regained independent mobility. Both the patient and his wife described audible grinding and clicking from the hip when walking. The patient reattended the ED three months later with a further deterioration in groin pain and difficulty mobilizing. X-rays were again interpreted as satisfactory and the patient was discharged. Pain and grinding from the hip persisted and the patient was referred by his GP for an orthopaedic opinion.

**Figure 1:**
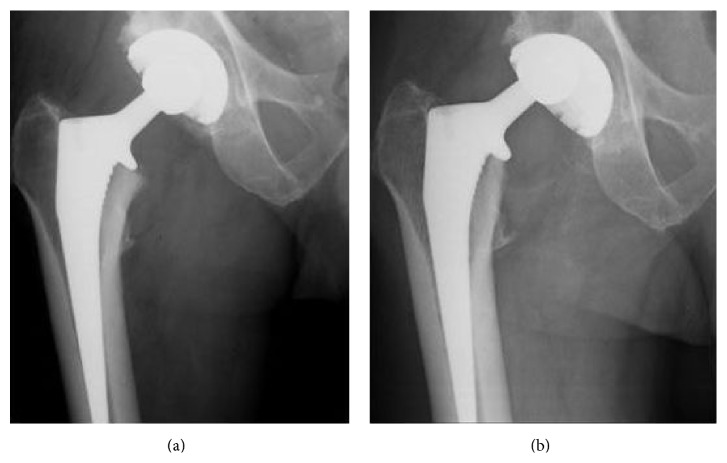
(a) Previous AP Hip X-ray demonstrates satisfactory component positioning. (b) AP Hip X-ray from ED attendance demonstrates acetabular liner dissociation. Comparison to (a) shows grossly eccentric superior migration of the femoral head within the acetabular shell.

**Figure 2:**
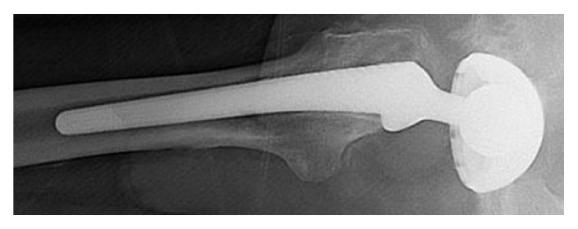
Lateral X-ray shows medial migration of the femoral head within the acetabular shell and excludes a dislocation as it confirms the prosthetic femoral head lies within the acetabular shell on a tangential view.

On review of X-rays from both previous ED attendances, there was evidence of acetabular liner dissociation (Figures 1 and 2). The patient was scheduled for urgent acetabular revision surgery.

At time of revision surgery, there was evidence of soft tissue metallosis with significant damage to the surfaces of both the femoral head and acetabular shell. The polyethylene liner had completely dissociated from the shell and lay within the adjacent soft tissues. Though acetabular shell orientation was satisfactory, concern over integrity of the locking mechanism necessitated revision. A 58 mm Pinnacle Sector shell with screw augmentation, a 58/32 mm Marathon highly cross-linked polyethylene liner, and a 32 mm metal Articul/eze femoral head were implanted (DePuy, Warsaw, IN, USA).

Postoperative recovery was uneventful and progress at time of last review was satisfactory.

## 3. Discussion

Acetabular liner dissociation is a serious but rare complication following THA that is specific to the modern uncemented acetabular component. The most recent National Joint Registry (NJR) Report shows that over 76,000 primary THA procedures were performed in England, Wales, and Northern Ireland in 2013, with uncemented acetabular components implanted in 65.4% of cases. Review of ten-year NJR data suggests an acetabular liner dissociation incidence of approximately 0.04% [1].

**Figure 3:**
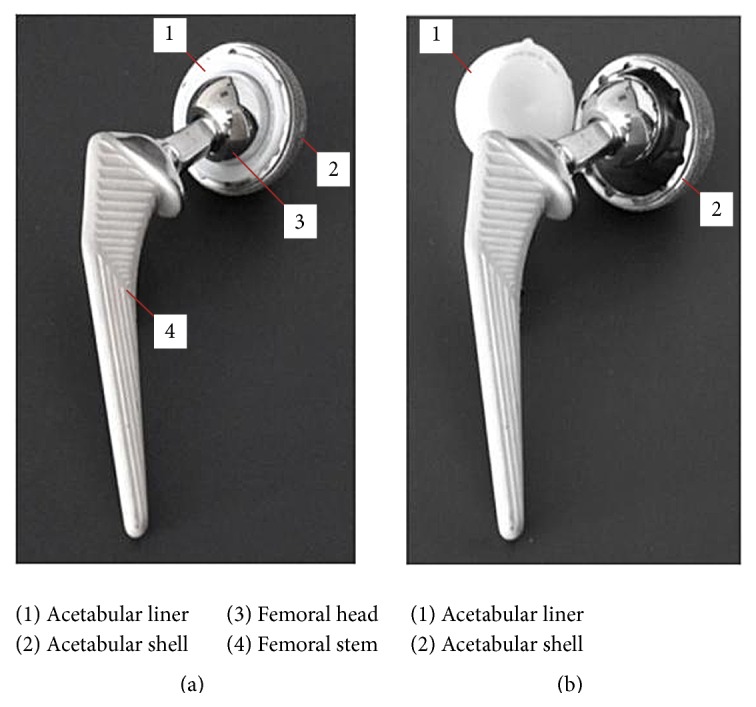
(a) Normal THA implant appearance. The polyethylene liner is well seated within the acetabular shell. (b) Acetabular liner dissociation appearance. The polyethylene liner has clearly migrated from its original position in (a).

The modern uncemented acetabular component is modular in design and consists of a metal shell which accepts a polyethylene or ceramic liner following insertion intraoperatively. Liner dissociation occurs due to failure of the locking mechanism between the metal shell and liner (Figure 3). It appears to be more commonly associated with polyethylene liners due to a difference in ceramic liner locking mechanism design [5, 6].

The aetiology of locking mechanism failure is believed to be related to shell geometry design and liner material properties in the adverse environment of increased torque or component impingement [7–9].

**Figure 4:**
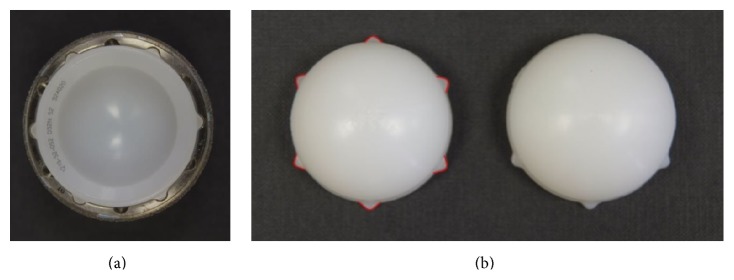
(a) Demonstrates shell Anti-Rotation Device (ARD) scallops which accept liner ARD tabs to enhance locking mechanism stability. (b) Normal appearance of polyethylene liner (6 ARD tabs highlighted in red) versus abnormal polyethylene liner in liner dissociation (failure of multiple ARD tabs).

In addition to a taper-lock mechanism, the Pinnacle shell design incorporates multiple Anti-Rotation Device (ARD) scallops which accept ARD tabs on the polyethylene liner in order to enhance stability (Figure 4(a)).

At time of acetabular component revision, multiple polyethylene liner ARD tabs were noted to have failed (Figure 4(b)).

Reports of both early and late liner dissociation are described in the literature and Joint Registries [1, 7–9]. It is likely that cases of early liner dissociation are more related to malseated components rather than true fatigue failure of the locking mechanism.

The senior author's routine practice includes checks to ensure correct liner seating and stability at time of initial implantation. Given the fact that the failure also occurred five years postoperatively, we believe the aetiology in this case was related to locking mechanism fatigue failure rather than initial component malseating.

Typical symptoms are of sudden onset of hip pain in a previously well-functioning prosthesis, followed by grinding or clicking with hip movements as the prosthetic femoral head articulates with the metal acetabular shell rather than the polyethylene liner [9, 10].

On examination, significant limb shortening or internal rotation as is commonly found with a posteriorly dislocated THA is unlikely. Audible grinding or clicking is reproduced with hip movements if pain permits.

AP Pelvic X-ray shows grossly eccentric superior migration of the femoral head within the acetabular shell [9]. Lateral X-ray shows medial migration of the femoral head within the acetabular shell and excludes a dislocation as it confirms the prosthetic femoral head lies within the acetabular shell on a tangential view. Comparison of previously normal X-rays on Picture Archived Communication Systems (PACS) can also be useful to aid the diagnosis if available (as shown in Table 1).

Appropriate management is orthopaedic referral for consideration of urgent acetabular component revision surgery.

Though acetabular liner dissociation is a rare complication following THA, increased awareness of the typical symptoms, signs, and radiological appearance will help avoid misdiagnosis.

A high index of clinical suspicion when assessing the painful THA is likely to improve patient outcomes and also reduce the risk of medical litigation.

## Figures and Tables

**Table 1:** Findings suggestive of liner dissociation in the acutely painful THA.

History	(i) Sudden onset of hip/groin pain in a previously well-functioning prosthesis (ii) New “grinding/clicking” noise from affected hip (iii) Difficulty fully weight bearing

Examination	(i) Audible “grinding/clicking” with passive hip movements (ii) Lack of significant limb shortening/rotation

Investigations	*AP Hip X-ray* Grossly eccentric superior migration of femoral head within acetabular shell *Lateral Hip X-ray* Medial migration of femoral head within acetabular shell. Excludes dislocation as confirms prosthetic femoral head lies within acetabular shell on a tangential view
